# Traditional Chinese medicine for treatment of sepsis and related multi-organ injury

**DOI:** 10.3389/fphar.2023.1003658

**Published:** 2023-01-19

**Authors:** Yaqin Song, Weiji Lin, Wei Zhu

**Affiliations:** ^1^ Department of Emergency Medicine, Tongji Hospital, Tongji Medical College, Huazhong University of Science and Technology, Wuhan, China; ^2^ Institute of Integrated Traditional Chinese and Western Medicine, Tongji Hospital, Tongji Medical College, Huazhong University of Science and Technology, Wuhan, China

**Keywords:** sepsis, inflammation, traditional Chinese medicine compounds, herbal monomer extracts, acupuncture

## Abstract

Sepsis is a common but critical illness in patients admitted to the intensive care unit and is associated with high mortality. Although there are many treatments for sepsis, specific and effective therapies are still lacking. For over 2,000 years, traditional Chinese medicine (TCM) has played a vital role in the treatment of infectious diseases in Eastern countries. Both anecdotal and scientific evidence show that diverse TCM preparations alleviate organ dysfunction caused by sepsis by inhibiting the inflammatory response, reducing oxidative stress, boosting immunity, and maintaining cellular homeostasis. This review reports on the efficacy and mechanism of action of various TCM compounds, herbal monomer extracts, and acupuncture, on the treatment of sepsis and related multi-organ injury. We hope that this information would be helpful to better understand the theoretical basis and empirical support for TCM in the treatment of sepsis.

## 1 Introduction

Sepsis is one of the leading causes of death from critical diseases, affecting each year more than 30 million people worldwide (Fleischmann et al., 2016). Sepsis is defined as a life-threatening organ dysfunction caused by the host’s dysfunctional response to infection, and is diagnosed based on a Sequential [Sepsis-related] Organ Failure Assessment (SOFA) score ≥2 (Singer et al., 2016). This definition highlights the importance of the systemic inflammatory response caused by immune disturbance. Pattern recognition receptors of innate immune cells recognize highly conserved microbial pathogen-associated molecular patterns (PAMPs), which activate signaling pathways, e.g., mitogen activated protein kinase (MAPK) and nuclear factor-κB (NF-κB), thus triggering the production and secretion of pro-inflammatory cytokines such as tumor necrosis factor alpha (TNF-α) and interleukin 1 (IL-1), IL-2, IL-6, and IL-8. These cytokines promote the adhesion between neutrophils and endothelial cells, which is followed by the activation of complement and blood coagulation cascades, and eventually lead to disseminated intravascular coagulation (Boyd et al., 2014; Huang et al., 2019). According to conventional view, after this early hyperinflammatory state, a subsequent hypo-inflammatory state partly from the release of anti-inflammatory cytokines such as IL-4 and IL-10, results in widely immune suppression. However, newer paradigms indicate a phase at which the pro-inflammatory and immunosuppression may occur simultaneously, and the complex interactions between host (genetics and comorbidities) and pathogen (type, virulence, and burden) is the leading factor (Cecconi et al., 2018).

Despite recent advances in anti-infective therapy and advanced life support, the high mortality rate of sepsis remains an urgent clinical challenge. High medical costs and possible sequela such as renal insufficiency and cognitive impairment make sepsis a global public health issue (Singer et al., 2016). In addition, one-sixth of survivors of sepsis frequently suffer from long-term impairments such as physical, cognitive, and organ function (Delano and Ward, 2016; Prescott and Angus, 2018). Accelerated progression of preexisting chronic conditions, residual organ damage, and impaired immune function accounts for the deterioration of health after sepsis (Prescott and Angus, 2018). Since a sustained, uncontrolled inflammatory response is regarded as key factor promoting the onset of sepsis and multiple organ damage, novel anti-inflammatory therapies are eagerly pursued for sepsis treatment. However, clinical trials aimed at blocking cytokine responses, such those testing TNF-α inhibitors and Toll-like receptor 4 (TLR4)/myeloid differentiation factor 2 (MD2) antagonists, failed to decrease the mortality rate of patients with septic (Fisher et al., 1996; Opal et al., 2013). In view of the limited treatment options, complementary and replacement therapies are increasingly investigated to develop new therapeutic measures to treat sepsis and related organ damage.

As an alternative and complementary therapy, TCM is increasingly recognized for its efficacy and safety in the treatment of diseases. For instance, in the last 2 years, TCM has been shown to improve immunity and alleviate fever and other symptoms of COVID-19 (Kang et al., 2022). Indeed, TCM offers unique advantages in the treatment of inflammatory diseases such as sepsis (classified as “exogenous fever disease” in TCM) (Li C et al., 2018; Lu Z.B. et al., 2020). According to TCM tenets, the main principles of sepsis treatment are clearing away heat and detoxifying, clearing the internal organs and expelling heat, promoting blood circulation and removing blood stasis, and strengthening the body and solidifying the detoxification. Many studies, including some clinical trials, have reported the effectiveness of TCM in suppressing inflammatory pathways, regulating the immune response, and inhibiting oxidative stress (Gao et al., 2019; Song Y et al., 2019; Xia et al., 2019; Shang et al., 2020). Evidence supports as well the potential of TCM compounds, herbal extracts, and electroacupuncture in the prevention and treatment of heart-, brain-, lung-, and intestine-related diseases (Kim et al., 2007; Liu et al., 2018; Nabavi et al., 2018; Pang et al., 2018; Zhang W et al., 2019; Zhang X et al., 2019; Liu S et al., 2020). Therefore, this review aims to summarize the efficacy and mechanism of action of TCM compounds, herbal extracts, and electroacupuncture, in the treatment of sepsis and sepsis-related multiple organ damage.

## 2 TCM compounds

TCM compounds are prescriptions consisting of two or more substances that provide multi-target synergistic effects (Sun et al., 2017). Through mutual compatibility of different chemical substances, components of TCM herbs react with each other, thereby lowering toxicity and adverse side effects and enhancing the therapeutic effects (Zhang R et al., 2019). Several TCM compounds, such as Xuebijing injection, Shenfu injection, Huanglian Jiedu decoction, Dachengqi decoction, and Xijiao Dihuang decoction, demonstrated efficacy in the treatment of sepsis-related organ injury.

### 2.1 Xuebijing injection

Xuebijing injection (XBJI) is an injectable prescription obtained from a combination of carthami flos, paeoniae radix rubra, szechuan lovage rhizome, angelicae sinensis radix, and salviae miltiorrhizae (Li et al., 2021) that shows distinct anti-inflammatory activities in several settings. XBJI decreased the expression of IL-6, TNF-α, IL-1β, and IL-12 in mouse macrophages stimulated by Pam3CSK4 (a synthetic tripalmitoylated lipopeptide mimicking bacterial lipoproteins) (Li T et al., 2020). Its pharmacology targets are the NF-κB and MAPK pathways, and its effects are manifested by inhibition of the phosphorylation of IKKα/β, IκBα, p65 NF-κB, and JNK (Li T et al., 2020). High-dose XBJI increased the number of T-regs, reduced the number of Th-17 T cells, downregulated the expression of inflammatory cytokines such as IL-6 and TNF-α, inhibited the infiltration of neutrophils in lung and kidney tissues, and improved survival in cecal ligation and puncture (CLP) model mice (Chen et al., 2018). In another study addressing also the mouse CLP sepsis model, XBJI administration significantly improved renal microvascular perfusion and oxygenation and inhibited renal expression of IL-1β, IL-6, TNF-α, and high mobility group box 1 (HMGB1) protein, although without affecting the survival rate (Liu J et al., 2021).

### 2.2 Shenfu injection

Shenfu injection (SFI) is mainly composed of ginsenosides and aconitine alkaloids (Liu et al., 2019). SFI was reported to exert antioxidant, anti-inflammatory, anti-apoptotic, and immunoregulatory effects in a rabbit model of lipopolysaccharide (LPS)-induced septic shock. SFI decreased serum levels of lactate dehydrogenase (LDH) and aminotransferase (AST), improved myocardial metabolism, and protected tissue morphology in the heart, liver, and kidney (Liu et al., 2019). SFI also suppressed inflammatory markers, such as TNF-α and IL-1β, in serum and heart of LPS-treated rats, and disrupted inflammatory signal transduction mediated by the mitogen-activated protein kinase repalmitoylated (MEK) and extracellular regulated protein kinase (ERK) pathways by decreasing p-MEK and p-ERK expression in LPS-stimulated H9C2 cells (Chen et al., 2020). Besides, SFI upregulated the expression of B cell lymphoma-2 (Bcl-2) and lowered the expression of Bid, t-Bid, and caspase-9, thus reducing cardiomyocyte apoptosis and attenuating myocardial injury in septic rats (Xu et al., 2020). In turn, beneficial effects of SFI on patients with septic shock were manifested by increased CD4^+^ and CD8^+^ T cells in peripheral blood, upregulated expression of human leukocyte antigen DR (HLA-DR) in monocytes, and enhanced cellular immunity (Zhang N et al., 2017). Importantly, clinical trials on patients with septic showed that SFI combined with conventional treatment led to significant improvement of clinical symptoms and prognosis, without obvious adverse reactions (Wen-Ting et al., 2012; Mo et al., 2014; Zhang Q et al., 2017; Wang X et al., 2019).

### 2.3 Shengmai injection

Shengmai injection (SMI), consisting of extracts from panax ginseng, ophiopogon japonicas, and schisandra chinensis, is one of the most widely used TCM prescriptions. According to the basic theory of TCM, simple tonic drugs like SMI cannot normally be used to treat infectious disease. However, in animal models of cardiac disease, marked improvement of myocardial metabolism was observed after treatment with SMI (Li et al., 2019; Cao et al., 2020). Specifically, SMI enhanced fatty acid and glucose oxidation, promoted mitochondrial biogenesis, and inhibited apoptosis by activating the AMPK signaling pathway in cardiomyocytes rendered hypertrophic by exposure to angiotensin II (Li et al., 2019). Meanwhile, SMI upregulated the expression of PTEN-induced kinase 1 (Pink1) and Parkin RBR E3 ubiquitin-protein ligase (Parkin), therefore improving myocardial mitophagy, in a mouse model of septic cardiomyopathy (Cao et al., 2020). Furthermore, SMI was shown to improve immune function and prolong survival in mice with CLP-induced peritonitis (Yu et al., 2005).

### 2.4 Huanglian Jiedu decoction

Huanglian Jiedu decoction (HLJDD) consist of rhizoma coptidis, radix scutellariae, cortex phellodendri, and fructus gardeniae and has been widely used in the treatment of inflammatory diseases (Lu Z et al., 2020). The beneficial regulatory role of HLJDD in lipid homeostasis represents the key mechanism of its anti-inflammatory actions. Upon LPS-induced inflammation in zebrafish, HLJDD ameliorated lipid imbalance mainly through the glycerophospholipid metabolism pathway. By normalizing the production of proinflammatory lipid intermediates, this effect was proposed to underlie TLR4/myeloid differentiation factor 88 (MyD88)/NF-κB pathway inhibition and reduced secretion of IL-6, IL-1β, TNF-α, and IFN-γ (Zhou et al., 2019). Notably, berberine, baicalin, and gardenin, the main components of HLJDD, were shown to play a protective role against sepsis-related multi-organ damage by binding to lipid A to neutralize LPS activity. This resulted in inhibition of IL-6, TNF-α, and IFN-γ secretion, as well as reduced synthesis of pathological lipid markers (Chen G et al., 2017). In a rat model of LPS-induced gingivitis, HLJDD administration suppressed serum inflammatory cytokines, lowered malondialdehyde (MDA) and reactive oxygen species (ROS) production, and upregulated total antioxidant capacity in periodontitis lysates. These effects were correlated with inhibition of AMPK and ERK1/2 expression (Zhang F et al., 2018).

### 2.5 Dachengqi decoction

Dachengqi decoction (DCQD) is composed of extracts from rheum palmatum l, magnolia henryi dunn, citrus aurantium l, and natrii sulfas. In a mouse model of LPS-induced acute lung injury (ALI), DCQD treatment inhibited TLR4/NF-κB signaling and IL-6, IL-8, and TNF-α secretion in lung tissue, and reduced pulmonary edema by upregulating aquaporin (AQP) 1/5 expression (Hu et al., 2019). Another study showed that DQCD attenuated intestinal vascular endothelial injury in rats with severe acute pancreatitis (SAP) induced by cerulein and LPS, and decreased matrix metalloproteinase 9 (MMP-9) and junctional adhesion molecule C (JAM-C) expression, while increasing AQP-1 expression, in TNF-α-treated vascular endothelial cells (Pan et al., 2017). Further *in vivo* and *in vitro* research on the SAP rat model indicated that DCQD inhibited the phosphatidylinositol 3-kinase (PI3K)/protein kinase B (AKT) signaling pathway, thereby inhibiting inflammation by decreasing production of IL-1β, IL-6, and TNF-α and promoting apoptosis of pancreatic acinar cells (Sun et al., 2020).

### 2.6 Xijiao Dihuang decoction

The Xijiao Dihuang decoction (XJDHD) comprises rehmannia, peony, cortex moudan, and cornu bubali and is used in China for the treatment of sepsis (Lu et al., 2020b). XJDHD administration was shown to improve the survival rate of rats subjected to CLP, as well as of cultured macrophages, by inhibiting aerobic glycolysis triggered by the TLR4/hypoxia-inducible factor 1α (HIF-1α)/pyruvate kinase M2 (PKM2) pathway (Lu et al., 2020b). Additional research on the above sepsis models further indicated that prolonged survival correlated with XJDHD-mediated inhibition of HIF-1a and p65 (Lu et al., 2020a).

## 3 TCM monomers

### 3.1 Triterpenoid

#### 3.1.1 Tanshinone IIA

Tanshinone IIA (TSA) occurs in the dried roots and rhizomes of salvia miltiorrhiza (lamiaceae), and is one of the main pharmacologically active components of hydrophilic tanshinones (Wang N et al., 2020). TSA has anti-inflammatory activity by inhibiting a variety of cytokines. In LPS-stimulated bone marrow-derived macrophages (BMDMs), TSA exposure inhibited succinate dehydrogenase (SDH)-mediated IL-1β and IL-6 production and blocked BMDM polarization towards the M1 phenotype (Liu Q.Y. et al., 2021). In addition, suggesting beneficial effects against neuroinflammatory and neurotoxic insults, TSA pretreatment was shown to attenuate pro-inflammatory cytokine secretion through inhibition of TLR4, MyD88, and TNF receptor associated factor 6 (TRAF6) expression and subsequent repression of signaling through the NF-κB and MAPK pathways in LPS-treated human U87 astrocytoma cells (Jin et al., 2020). TSA treatment also reduced calcium inflow, inhibited transient receptor potential melastatin 7 (TRPM7), and suppressed the release of pro-inflammatory cytokines in pulmonary interstitial macrophages from rats with sepsis-induced ALI (Li J et al., 2018). Meanwhile, incubation with Salvia miltiorrhiza extract decreased LPS-induced phosphorylation of IκB-α and IKK, thus inhibiting NF-κB activity, inhibited MAPK phosphorylation, and disrupted TLR4 dimerization to prevent TLR4-MyD88 complex formation in RAW264.7 cells (Gao et al., 2017).

#### 3.1.2 Astragaloside IV

Astragaloside IV (AS-IV) is a small saponin obtained from the roots of astragalus membranaceus (Xia et al., 2020). AS-IV has anti-inflammatory and neuroprotective activities. Studies in the CLP mouse model showed that AS-IV treatment inhibited the secretion of inflammatory cytokines and downregulated the expression of NLRP3, apoptosis associated speck like protein containing CARD (ASC), and cleaved caspase-1 in both intestinal tissue and BALF, thus reducing multi-organ injury (Huang and Li, 2016; Xie et al., 2020). AS-IV administration was also shown to protect blood-brain barrier integrity in LPS-treated mice by upregulating zonula occludens-1 (ZO-1) and occludin and downregulating vascular cell adhesion molecule 1 (VCAM1) expression in brain micro-vessels; experimental results in cultured bEnd.3 and microglial cells attributed this protective effect to both activation of the Nrf2-dependent antioxidant response and inhibition of the NF-κB/NLRP3 inflammasome signaling pathway (Li H et al., 2018; Yang et al., 2019). Further research showed that AS-IV attenuated LPS-induced neuroinflammation in mice by upregulating the expression of PPARγ and inducing phosphorylation of glycogen synthase kinase-3β (GSK3-β) in the hippocampus. AS-IV-mediated neuroprotection was further evidenced by reduced expression of inflammatory factors, in association with NF-κB signaling suppression (Song et al., 2018). In a mouse model of acute *E. coli* peritoneal infection, AS-IV treatment alleviated peritonitis symptoms by promoting the influx of neutrophils to the infection site, an effect mediated by inhibition of G protein-coupled receptor kinase-2 (GRK2) expression and subsequent blockade of LPS-induced suppression of CXC motif chemokine receptor 2 (CXCR2) on neutrophils (Huang et al., 2016). Moreover, in LPS-treated rats, AS-IV administration attenuated cardiac dysfunction, reduced myocardial damage, improved mitochondrial energy metabolism, and inhibited cardiomyocyte apoptosis and autophagy by downregulating miRNA-1 expression (Wang et al., 2021).

#### 3.1.3 Glycyrrhizin

Glycyrrhizin is a natural triterpene glycoside and the major active component of gan cao (licorice root). Glycyrrhizin treatment was associated with significantly improved survival in a rat model of CLP-induced sepsis. This effect was associated with suppression of HMGB1 expression and inhibition of downstream MAPK/NF-κB pathways both *in vivo* and *in vitro* (Zhao et al., 2017). In turn, in LPS-treated mice, a protective role of glycyrrhizin on heart and lung was attributed to the increase in the ratio of myeloid-derived suppressor cells (MDSCs) to CD11b^+^Gr1 bone marrow cells in the blood, heart, and lungs (Seo et al., 2017). In addition, glycyrrhizin was shown to negatively regulate PI3K/mTOR signaling and inhibit the expression of inflammatory markers such as iNOS, COX-2, HMGB1, TNF-α, IL-1β, and IL-6 in LPS-stimulated human liver macrophages (Shen et al., 2020).

#### 3.1.4 Triptolide

Triptolide is a primary bioactive ingredient of the roots of the Chinese herb tripterygium wilfordii Hook. F. Triptolide exerted vascular anti-inflammatory effects by downregulating proinflammatory cytokine and chemokine secretion, attenuating VCAM-1 and intercellular adhesion molecule-1 (ICAM-1) expression, and inhibiting IκBα phosphorylation and NF-κB p65 DNA binding activity in LPS-stimulated endothelial cells (Song C et al., 2019). ZT01, a triptolide derivative, showed also anti-inflammatory activity *via* reducing TNF-α and IL-6 levels, blocking the formation of the TGF-β-activated kinase1 (TKA1)/TAK1-binding protein1 (TAB1) complex, and inhibiting the phosphorylation of both mitogen-activated protein kinase kinase 4 (MKK4) and JNK in both *in vivo* and *in vitro* sepsis models (Fu et al., 2020).

#### 3.1.5 Artemisinin

Artemisinin is a sesquiterpene lactone obtained from the sweet wormwood plant *Artemisia annua*. Although mainly recognized by its efficacy to treat malaria, there is growing evidence that artemisinin may be useful to treat several health conditions, including sepsis, by exerting potent anti-inflammatory and immunoregulatory effects. Artemisinin treatment was shown to significantly reduce LPS-induced cognitive impairment in mice by attenuating both neuronal damage and microglial activation in the hippocampus (Lin S.P et al., 2021). This study further showed that artemisinin reduced TNF-α, IL-6, IL-1α, IL-1β, and iNOS production and suppressed the migratory ability of LPS-stimulated BV2 microglial cells by activating the AMPKα1 pathway and inhibiting nuclear translocation of NF-κB (Lin S.P et al., 2021). Artesunate is a water-soluble derivative of artemisinin with multiple biological activities. In rats with LPS-induced ALI, artesunate treatment reduced TNF-α and IL-6 levels in BALF, decreased oxidative stress markers (i.e., MDA, SOD, and GSH-Px), and reduced apoptosis of lung cells by activating the mTOR/AKT/PI3K signaling pathway (Zhang E et al., 2020). Notably, artesunate was reported to reverse sepsis-induced immunosuppression in mice by interacting with the vitamin D receptor in an autophagy- and NF-κB-dependent manner (Shang et al., 2020).

#### 3.1.6 Ginsenoside Rg1

Ginsenoside Rg1 (GRg1), a triterpenoid saponin, is the main bioactive component of ginseng (panax ginseng). Administration of GRg1 ameliorated LPS-induced acute myocardial injury by inhibiting the NF-κB pathway and attenuating inflammatory responses, including NLRP3 expression (Luo et al., 2020). GRg1 treatment also alleviated lung injury and extended survival in mice with LPS-induced ALI; experiments in LPS-challenged pulmonary epithelial A549 cells further showed that GRg1 exposure inhibited ROS production, prevented apoptosis, and reduced ER stress and inflammatory cytokine expression by upregulating Sirt1 (Wang Q.L. et al., 2019). In animal models of sepsis-associated ALI and encephalopathy, the protective effect of GRg1 was shown to also depend on autophagy enhancement, *via* a mechanism related to the activation of Nrf-2 and inhibition of NF-κB signal transduction (Li Y et al., 2017; Ji et al., 2021).

### 3.2 Flavonoids

#### 3.2.1 Apigenin

Apigenin, a flavonoid found in abundance in many fruits and vegetables, has shown remarkable efficiency in controlling the inflammatory response. In LPS-treated mice, apigenin administration decreased the levels of cardiac troponin I (cTnI), cardiac myosin light chain-1 (cMLC1), and inflammatory cytokines such as TNF-α, IL-1β, MIP-1α, and MIP-2, an effect attributed to reduced NF-κB nuclear translocation and enhanced peroxisome proliferator-activated receptor gamma (PPARγ) nuclear translocation (Li F et al., 2017; Zhou et al., 2017). In addition, apigenin ameliorated LPS-induced acute liver injury in mice *via* inhibiting oxidative stress, evidenced by upregulation of the activities of superoxide dismutase (SOD), catalase (CAT), glutathione-S-transferase (GST) and glutathione reductase (GR). This protective role was partly dependent on increased hepatic expression of nuclear factor erythroid 2-related factor 2 (Nrf-2) (Zhou et al., 2017). The potential of apigenin to counteract neuroinflammation was in turn suggested by its ability to downregulate the expression of CD68 (an M1 pro-inflammatory microglia marker), OX42 (a microglia activation marker), IL-6, and glycoprotein 130 in LPS-stimulated neuronal/glial co-cultures (Dourado et al., 2020).

#### 3.2.2 Salidroside

Salidroside is extracted mainly from the root and rhizome tissues of the rose (rhodiola rosea). Many studies reported that salidroside has anti-inflammatory, anti-oxidative, and antibacterial properties. In rats with myocardial or lung injury triggered by LPS-induced endotoxemia, salidroside enhanced antioxidative activity and inhibited iNOS and COX2 expression, as well as NF-κB phosphorylation, in cardiac and lung tissues (Chen L et al., 2017; Jingyan et al., 2017; Zheng et al., 2020). Salidroside also reduced lung inflammation and alleviated ALI symptoms by upregulating Sirt1 expression and inhibiting both NF-κB activity and nucleocytoplasmic translocation of HMGB1 both *in vivo* and *in vitro* (Lan et al., 2017; Qi et al., 2017). In LPS-stimulated HUVECs, salidroside exposure increased antioxidant activity, inhibited apoptosis and NLRP3 inflammasome activation, and decreased the expression of NLRP3-related proteins, including ASC and caspase-1 (You et al., 2021).

#### 3.2.3 Baicalein

Baicalein is a flavonoid extracted from the roots of the Chinese herb scutellaria baicalensis georgi. Its anti-inflammatory and anti-oxidative qualities have been demonstrated in several experimental settings. Baicalein-mediated protection against neuroinflammation was exemplified by its ability to downregulate LPS-induced NO generation, inhibit the expression of inflammatory cytokines such as IL-6, TNF-α, and COX2, and suppress NF-κB and p65-MAPK signaling in BV2 microglial cells and macrophages (Luo et al., 2017; Yan et al., 2020). Baicalin alleviated LPS-induced liver injury in mice by inhibiting the expression of IL-1α, IL-1β, and gasdermin D (GSDMD) and blocking NLRP3/IL-1β signaling (Xiao et al., 2021). Moreover, baicalin prevented the development of LPS-induced ALI and alleviated colitis symptoms by blocking LPS-induced TLR4/MD-2 complex formation, inhibiting the activation of MAPK and NF-κB signaling pathways, and reducing leukocyte infiltration and production of inflammatory mediators (Luo et al., 2017; Chen H et al., 2019).

### 3.3 Phenols

#### 3.3.1 Resveratrol

Resveratrol is a non-flavonoid polyphenol with potent antioxidant properties, present in the skin of fruits such as grapes and berries. Resveratrol pretreatment protected mice against CLP-induced ALI. Research on LPS stimulated MH-S alveolar macrophages suggested that the underlying mechanism was related to inhibition of NF-κB and MAPK pathways, as well as of LPS-induced autophagy, which depended on enhanced expression of vascular endothelial growth factor B (VEGF-B) (Yang et al., 2018). In primary monocytes, resveratrol inhibited the LPS-stimulated inflammatory response by blocking phospholipase D activity and its downstream signaling molecules SphK1, ERK1/2, and NF-κB, and protected mice against septic shock induced by CLP (Wang B et al., 2020). In rats subjected to CLP, resveratrol improved vasodilation and hemodynamic parameters by upregulating endothelial nitric oxide synthase (eNOS) and downregulating iNOS, Rac family small GTPase 1 (RAC-1), and HIF-1α expression in arterial tissue (Zhang Z.S. et al., 2019). Resveratrol was also shown to ameliorate acute kidney injury (AKI) induced by sepsis by inhibiting renal inflammation triggered by endoplasmic reticulum (ER) stress activated-IRE1/NF-κB pathway activation (Wang et al., 2017). In LPS-treated mice, cardioprotective effects of resveratrol, evidenced by decreased 4-hydroxynonenal and MDA levels in myocardial tissue and improved contractility and Ca^2+^ homeostasis in cardiomyocytes, were attributed to increased Nrf-2 expression and to phospholamban oligomerization leading to enhanced SERCA2a activity (Bai et al., 2016).

#### 3.3.2 Paeonol

Paeonol, a phenolic compound found in peonies such as paeonia suffruticosa (moutan cortex), shows multiple pharmacological effects, including anti-inflammatory and anti-tumoral activities. Paeonol exposure increased the expression of miR-339-5p and downregulated the expression of inflammatory markers, such as TNF-α, IL-1β, IKK-β, and HMGB1, in LPS-stimulated RAW264.7 cells (Mei et al., 2019). In a rat model of sepsis-induced AKI, paeonol treatment showed protective effects by lowering serum levels of TNF-α and IL-1β and suppressing NF-κB signaling in renal tissue (Mei et al., 2019). In the advanced stage of sepsis, impaired phagocytic activity of macrophages and monocytes contributes to immune dysfunction. Interestingly, the decline in the phagocytic ability of peritoneal macrophages induced by LPS could be reversed by paeonol co-administration, an effect mediated by suppression of HMGB1 nucleocytoplasmic translocation (Miao et al., 2020).

#### 3.3.3 6-gingerol

6-gingerol, a major polyphenol extracted from the ginger rhizome (zingiber officinale roscoe), exhibits anti-inflammatory, antioxidant, anticancer, and neuroprotective properties. Treatment with 6-gingerol suppressed systemic IL-1β release and prolonged survival in mice with CLP-induced sepsis. Complementary *in vitro* studies showed that 6-gingerol pre-treatment blocked MAPK-dependent NLRP3 inflammasome activation in LPS/ATP-treated BMDMs and RAW264.7 cells, which attenuated pyroptosis and therefore decreased the release of mature IL-1β into the medium (Zhang F.L. et al., 2020). In mice with CLP-induced acute liver injury, 6-gingerol administration reduced serum levels of AST, ALT, and IL-1β and elicited antioxidant and anti-apoptotic effects by upregulating hepatic Nrf-2 and HO-1 transcription (Hong et al., 2020). Similarly, renoprotective effects of 6-gingerol, consistent with its antioxidant and anti-inflammatory properties, were reported in a rat model of sepsis-induced AKI (Rodrigues et al., 2018).

### 3.4 Alkaloids

#### 3.4.1 Berberine

Berberine is potent anti-inflammatory alkaloid compound extracted from herbs such as cortex phellodendri and rhizoma coptidis. In animal studies, berberine treatment ameliorated sepsis-related intestinal vascular barrier injury by modulating the Apoliprotein M (ApoM)/Sphingosine-1-Phosphate (S1P) axis and the Wnt/β-catenin pathway (He et al., 2018; Li Y et al., 2020). In LPS-induced acute respiratory distress syndrome (ARDS), berberine treatment prevented endothelial glycocalyx damage and hence reduced pulmonary vascular permeability by inhibiting TNF-α, IL-1β, IL-6, MMP-9, and heparanase expression, attenuating ROS production, and decreasing neutrophil infiltration in BALF (Huang et al., 2018). Further research in the ARDS model indicated that berberine’s protective mechanism resulted from inhibition of TLR4/NF-κB and JAK2/STAT3 signaling pathways (Xu et al., 2021). Cardioprotective effects of berberine, manifested by improved cardiac diastolic function and hemodynamics, were observed in rats with LPS-induced septic cardiomyopathy (Chen et al., 2021). Berberine treatment was also reported to reduce LPS-induced cognitive deficits and restore spatial learning ability in rats. These effects were correlated with increased antioxidant activity, reflected by upregulation of glutathione peroxidase (GPx), SOD, CAT, and glutathione, and decreased acetylcholinesterase (AChE), MDA, carbonyl protein, and caspase-3 activity in the hippocampus (Sadraie et al., 2019). In cultured RAW264.7 macrophages, berberine exposure inhibited LPS-induced synthesis of proinflammatory cytokines (MCP-1, IL-6, and TNF-α). This effect was mediated by reversal of LPS-induced Sirtuin1 (Sirt1) downregulation, which inhibited IκΒα degradation and IKK phosphorylation, effectively suppressing NF-κB signaling (Zhang H et al., 2017).

#### 3.4.2 Cordycepin

Cordycepin is the main active component of the fruiting bodies of the ascomycete fungus cordyceps militaris. In a mouse model of LPS-induced ALI, cordycepin administration downregulated the expression of MPO and MDA in lung tissue and reduced TNF-α and IL-1β levels in BALF. The underlying mechanism was found to be related to inhibition of NF-κB activity and stimulation of Nrf-2 and HO-1 expression (Lei et al., 2018; Qing et al., 2018). Cordycepin was also reported to decrease LPS-induced pro-inflammatory cytokine production and COX-2 expression in RAW264.7 and THP-1 cells, effects attributed to the inhibition of the NLRP3 inflammasome and the ERK1/2 signaling pathway (Yang J et al., 2017).

### 3.5 Quinones

Emodin belongs to the Quinones class of compounds and is mainly extracted from the dry roots and rhizome of rhubarb (Hu et al., 2020). According to TCM precepts, emodin is effective in reducing accumulation, cooling blood, reducing fire, promoting blood circulation, removing blood stasis, and draining the gallbladder to relieve jaundice. Emodin was shown to possess a wide range of pharmacological properties, linked to anticancer, hepatoprotective, anti-inflammatory, antioxidant, and antimicrobial effects (Cui et al., 2020). In rats with LPS-induced ALI, emodin suppressed IKKβ, p-IKKβ, p65, and p-p65 levels, decreased NF-κB DNA binding activity, and inhibited IL-8, IL-1β, TNF-α, and myeloperoxidase (MPO) expression in lung tissues, and increased the proportion of Gr1^+^/CD11b^+^ cells in bronchoalveolar lavage fluid (BALF) (Liu B et al., 2020). It was reported that emodin further protected against ALI by downregulating the mechanistic target of rapamycin kinase (mTOR)/HIF-1α/vascular endothelial growth factor (VEGF) signaling pathway (Li X et al., 2020). Granulocytes are the first line of defense against pathogen invasion and play a crucial role in innate immunity. Emodin could upregulate the ability of granulocytes to phagocytize bacteria and generate of neutrophil extracellular trap (NETs), meanwhile, downregulated the production of ROS expression in the LPS-stimulated granulocytes, therefore alleviating lung tissue damage (Mei et al., 2020). Anti-neuroinflammatory effects of emodin were also evidenced by decreased TNF-α, IL-6, nitric oxide (NO), prostaglandin E2 (PGE2), inducible nitric oxide synthase (iNOS), and cyclooxygenase 2 (COX-2) synthesis, inhibition of NF-κB and activation of activator protein-1 (AP-1) signaling pathways, and suppressed phosphorylation of STATs and MAPKs in LPS-stimulated microglial cells (Park et al., 2016; Xie et al., 2019). In a mouse model of LPS-induced acute liver injury, emodin treatment alleviated hepatic inflammation and promoted M2 polarization of liver macrophages. Consistent with these findings, emodin exposure downregulated TLR4, MyD88, Toll/interleukin-1 receptor (TIR) domain-containing adaptor protein (TIRAP), TRAF-6, TIR-domain-containing adapter-inducing interferon-β (TRIF), interferon regulatory factor 3 (IRF-3), and AP-1 protein expression in LPS-activated RAW264.7 macrophages (Ding et al., 2018). In a mouse model of LPS-induced septic cardiomyopathy, emodin treatment inhibited expression of cardiac injury markers, i.e., LDH and creatine kinase-MB (CK-MB), and downregulated the expression of inflammatory cytokines. These protective effects were attributed to inhibition of NOD-like receptor family, pyrin domain containing 3 (NLRP3) inflammasome activation in cardiomyocytes (Dai et al., 2021).

## 4 Acupuncture

Acupuncture is a non-pharmacological TCM method of treating diseases that acts through mechanical stimulation (usually by needling, but also *via* heat or pressure) on specific skin sites (acupoints) that lie along passageways through which energy flows throughout the body (meridians). In recent years, evidence that acupuncture on specific acupoints, i. e. Zusanli (ST36) and Tianshu (ST25), can regulate immunity suggested that this procedure represents a promising alternative in clinical anti-inflammatory therapy (Kim et al., 2007; Liu S et al., 2020). As a non-pharmacological therapy, and supported by preclinical studies, acupuncture has increasingly attracted the attention of clinicians (Lai et al., 2020; Liu S et al., 2020). Electroacupuncture treatment was shown to improve sepsis-related damage of brain, heart, kidney, intestines, and other organs in animal models. Its application improved survival rate in CLP model rats, and ameliorated cognitive impairment in rats with sepsis-related encephalopathy by inhibiting hippocampal synaptic damage, neuronal loss, oxidative stress, and release of inflammatory cytokines trough activation of the Nrf-2/HO-1 pathway and hippocampal α7 nicotinic acetylcholine receptors (Han et al., 2018; Li C et al., 2020). In a rat model of sepsis, electroacupuncture pretreatment at ST36 attenuated inflammatory responses, decreased plasma urea, creatinine, and D-lactate levels, and increased intestinal tight junction protein occludin expression and intestinal barrier permeability, thereby reducing acute kidney and intestinal injury (Zhu et al., 2015; Zhang Z et al., 2018; Harpin et al., 2020). In the rat CLP model, electroacupuncture treatment at ST 36 reduced the activity of plasma CK-MB and offered cardioprotection by reducing TNF-α, NO, MPO levels and water content in myocardial tissue by activating the cholinergic anti-inflammatory pathway (Zhang L et al., 2018). In clinical trials, electroacupuncture at both ST36 and Shangjuxu (ST37) significantly reduced procalcitonin (PCT), TNF-α, intestinal fatty acid-binding protein (I-FABP), D-lactate, citrulline, and the TCM quantitative score of intestinal dysfunction in patients with sepsis-related intestinal dysfunction and intestinal obstruction (Meng J.B. et al., 2018). In addition, a prospective randomized controlled trial found that electroacupuncture at both ST 36 and Guanyuan (RN 4) in patients with sepsis played an immunoprotective role by reducing the APACHE II score, increasing CD3^+^, CD4^+^, CD8^+^ expression and the CD4^+^/CD8^+^ ratio, and increasing HLA-DR expression in lymphocytes (Yang et al., 2016).

**FIGURE 1:**
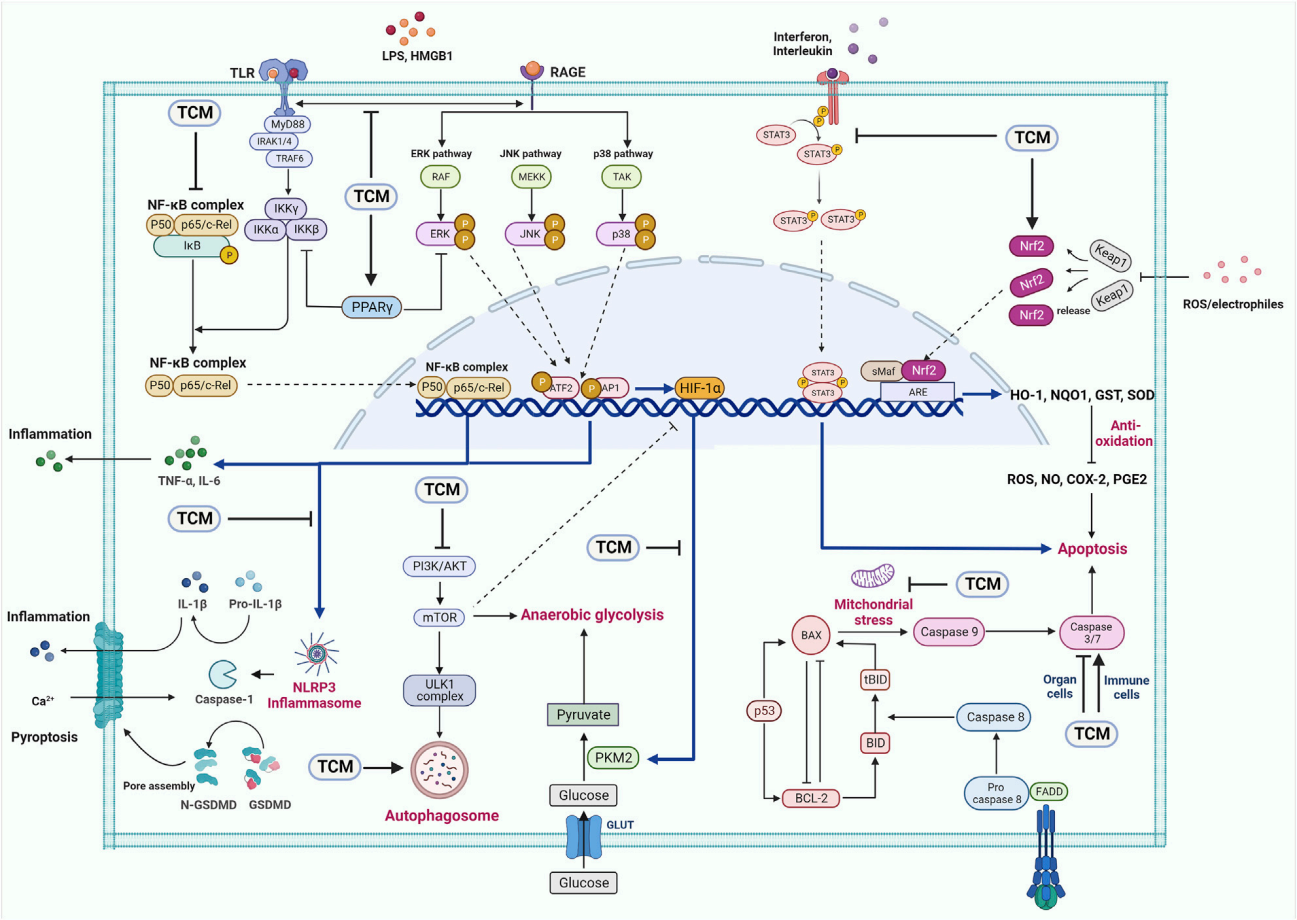
The potential applications of TCM in sepsis. TCM can protect sepsis through TLR4/NF-κB, PI3K/mTOR, NLRP3 inflammasome, MAPK and other signaling pathways.

The above systematically summarized the research and mechanism of TCM in the treatment of sepsis (the relevant mechanisms are shown in Figure 1). Tables 1, 2 respectively summarize the relevant research of TCM compounds and monomers in the treatment of sepsis in recent years, which further prove the effectiveness of TCM.

**TABLE 1:** Characteristics of TCM compounds on sepsis.

Compound	Efficacy	Composition	Animal/Cell model	Dose/Concentration	Targets	Ref
Xuebijing Injection	Regulating the balance of Tregs and Th17 cells; Decreasing inflammatory mediators and bacterial load	Honghua (Carthami tinctorii L.), Chishao (Paeonia lactiflora Pall.), Chuanxiong (Ligusticum chuanxiong Hort.), Danggui [Angelica sinensis (Oliv.) Diels], and Danshen (Salvia miltiorrhizae Bge.)	Mice: MRSA 7 × 10^8^ CFU, RAW264.7 cells: Pam3CSK4 100 ng/mL (Li T et al., 2020); Mice: CLP (Chen et al., 2018); Rats: CLP (Liu J et al., 2021)	Mice: 5, 10 mL/kg, RAW264.7 cells: 3, 10, 30 μL/mL (Li T et al., 2020); Mice:18 mL/kg (Chen et al., 2018); Rats: 4 mL/kg (Liu J et al., 2021)	NF-κB and MAPK↓; PI3K/Akt phosphorylation ↓; HMGB1 ↓	Chen et al. (2018); Li T et al. (2020); Liu J et al. (2021)
Shenfu injection	Improving energy metabolism and antioxidation; Attenuating the inflammation and apoptosis	Ginsenosides and aconitine alkaloids	Rabbits: LPS .6 mg/kg (Liu et al., 2019); Rats: LPS 20 mg/kg, H9C2 cells: LPS 16 μg/mL (Chen et al., 2020); Mice: LPS 8 mg/kg (Xu et al., 2020)	Rabbits: 4.5, 6, 8 mL/kg (Liu et al., 2019); Rats: 10 mL/kg, Cells: 80 μL/mL (Chen et al., 2020); Mice: 3, 10 mL/kg (Xu et al., 2020)	p-MEK ↓, p-ERK ↓; cleaved-caspase 3 ↓, caspase 9↓, Bax↓; Bid and t-Bid ↓; Bcl-2 ↑	Chen et al. (2020); Liu et al. (2019); Xu et al. (2020)
Shengmai injection	Promoting myocardial mitochondrial autophagy and mitochondrial membrane potential	Panax ginseng, Ophiopogon japonicas and Schisandra chinensis	Mice: LPS 8 mg/kg, HL-1 cells: LPS 1 μg/mL	Mice: 10 mL/kg	caspase-3/Beclin-1axis ↓	Cao et al. (2020)
Huanglian Jiedu decoction	Anti-inflammatory	Coptidis Rhizoma, Scutellariae Radix, Phellodendri Chinensis Cortex, and Gardeniae Fructus	Zebrafishes: LPS 10 mg/mL	Zebrafishes: 50 μg/mL	TLR4/MyD88 ↓	Zhou et al. (2019)
Dachengqi decoction	Alleviating the release of inflammatory cytokines and regulating capillary permeability	Da Huang, Houpu, Zhishi, and Mangxia	Rats: LPS 10 mg/kg, HUVEC-5a cells: LPS 100 ng/mL	Rats: .9 g/kg, HUVEC-5a cells: 100 μg/mL	TLR4 ↓; NF-κB ↓	Hu et al. (2019)
Xijiao Dihuang decoction	Inhibiting aerobic glycolysis and inflammatory cytokines	Rehmannia, Peony, Cortex Moudan and Cornu Bubali	Rats: CLP, NR8383 cells: LPS 1 μg/mL (Lu et al., 2020a; Lu et al., 2020b)	Rats: 12.5, 25 g/kg, NR8383 cells: 4 mg/mL (Lu et al., 2020b); Rats: 25 g/kg, NR8383 cells: 4 mg/mL (Lu et al., 2020a)	TLR4/HIF-1α/PKM2 ↓; NF-κB ↓; HIF-1α ↓	Lu et al. (2020a); Lu et al. (2020b)
Liang-Ge-San	Inhibiting inflammatory response; Reducing infiltration of inflammatory cells; Decreasing recruitment of macrophages and neutrophils	Fructus forsythiae (Lian Qiao), Rheum officinale (Da Huang), Fructus Gardeniae (Zhi Zi), Radix Scutellariae (Huang Qin), Liquorice (Gan Cao), Mint (Bo He), Mirabilite (Mang Xiao)	Zebrafishes larvae: LPS .5 mg/mL, RAW264.7 cells: LPS 100 ng/mL	Zebrafish: 62.5, 125, 250 μg/mL, RAW264.7 cells: 25, 50, 100 μg/mL	p-JNK ↓; p-Nur77 ↓	Zhou et al. (2020)
Xuanbai Chengqi decoction	Attenuating proinflammatory cytokines release	Rheum palmatum rhizome and root (Dahuang), Gypsum Fibrosum (Shigao), Prunus armeniaca seed (Kuxingren), and *Trichosanthes kirilowii* fruit (Gualou)	Rats: LPS 8 mg/kg	Rats: 5, 20 g/kg	PI3K/mTOR/HIF-1α/VEGF↓	Zhu et al. (2021)
Sini decoction	Inhibiting inflammatory cell infiltration and the production of inflammatory cytokines; Antioxidant stress	Aconite, Liquorice and Ginger Rhizome	Mice: LPS 8 mg/kg, HUVECs cells: LPS 1 μg/mL	Mice: 5 g/kg, HUVECs cells: 6, 12.5, 25 mg/mL	MAPK↓, ACE/AT1R↓; ACE2/Ang1–7↑	Chen Q et al. (2019)
Fangji Fuling decoction	Inhibiting inflammatory reaction and apoptosis	Stephania tetrandra S.Moore (Fangji), *Astragalus propinquus* Schischkin (Huangqi), Cinnamomum cassia (Nees & T.Nees) J.Presl (Guizhi), *Glycyrrhiza* uralensis Fisch. (Gancao), and Poria cocos (Schwein.) F.A.Wolf (Fuling)	Mice: LPS 10 mg/kg, HK-2 cells: LPS 1 μg/mL	Mice: 25,50 mg/kg, HK-2 cells: 200 μg/mL	iNOS↓; NF-κB↓	Su et al. (2018)
Xuefu Zhuyu decoction	Inhibiting apoptosis; antioxidation	Prunus persica (L) Batch. (Tao Ren), Angelicae sinensis (oliv.) Diels. (Dang Gui), Ligusticumi chuangxiong Hort. (Chuang Xiong), Carthamus tinctorius L. (Hong Hua), Paeonia lactiflora Pall. (Chi Shao), Rehmannia glutinosa Libosch. (Di Huang), Citrus aurantium L. (Zhi Qiao), Bupleurum chinense DC. (Chai Hu), *Platycodon grandiflorum* (Jacq) A. DC. (Jie Geng), *Achyranthes bidentata* BL. (Niu Xi), *Glycyrrhiza* uralensis Fisch. (Gan Cao)	Mice: LPS 10 mg/kg	Mice: 3.9, 7.8, 15.6 g/kg	SOD↑, Bcl-2↑; TNF-α ↓, IL-1β ↓, IL-6 ↓; MDA ↓, Bax ↓, Caspase-3↓	Meng F et al. (2018)
Lianhua Qingwen	Inhibiting Apoptosis	Forsythia suspensa (Thunb.) Vahl (Lianqiao, LQ), *Lonicera japonica* Thunb. (Jinyinhua, JYH), Ephedra sinica Stapf (Mahuang, MH), Isatis tinctoria L. (Banlangen, BLG), Pogostemon cablin (Blanco) Benth. (Guanghuoxiang, GHX), Rheum palmatum L. (Dahuang, DH), *Glycyrrhiza* uralensis Fisch. (Gancao, GC), Dryopteris crassirhizoma Nakai (Mianmaguanzhong, GZ), Rhodiola crenulata (Hook.f. and Thomson) H. Ohba (Hongjingtian, HJT), Houttuynia cordata Thunb. (Yuxingcao, YXC), Prunus sibirica L. (Kuxingren, KXR), Gypsum and l-Menthol	Mice: LPS 5 mg/kg	Mice: .7, 1.4, 2.8 g/kg	Bcl-2↑; Bax↓, caspase-3↓, caspase-9↓; p53↓	Yang et al. (2021)

**TABLE 2:** Characteristics of TCM extracts/monomers on sepsis.

Monomers	Molecular structure	Effects	Source	Animal/Cell model	Dose/Concentration	Targets	Ref
Tanshinone IIA	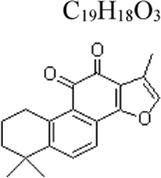 C_19_H_18_O_3_	Anti-inflammatory; Antifibrotic; Antioxidative activities	Salvia miltiorrhiza Bunge	Mice: LPS 15 mg/kg, RAW264.7 cells: LPS 100 ng/mL (Liu Q.Y. et al., 2021); U87 cells: LPS 100 ng/mL (Jin et al., 2020)	Mice: 20 mg/kg, RAW264.7 cells: 10 μM(Liu Q.Y. et al., 2021); U87 cells: 1, 5, 10 μM(Jin et al., 2020)	Sirt2 ↑ HIF-1α ↓, SDH↓; NLRP3 ↓, HK-II ↓ PKM2 ↓; TLR4/NF-κB/MAPKs↓	Liu Q.Y. et al. (2021)
							Jin et al. (2020)
Astragaloside IV	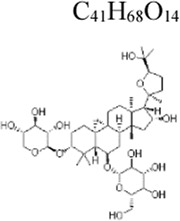 C_41_H_68_O_14_	Anti-inflammatory; Anti-oxidative and anti-apoptotic effects; Decreasing barrier permeability; Increasing tight junction	*Astragalus membranaceus* (Fisch) Bge	Mice: CLP, Caco-2 cells: LPS 100 μg/mL (Xie et al., 2020); Rats: CLP (Huang and Li, 2016); Mice: LPS 1 mg/kg (Song et al., 2018)	Mice: 3 mg/kg Caco-2 cells: 200 μg/mL (Xie et al., 2020); Rats: 2.5, 5, 10 mg/kg (Huang and Li, 2016); Mice: 20, 40 mg/kg (Song et al., 2018)	RhoA/NLRP3 ↓ PPARγ ↑ NF-κB ↓	Xie et al. (2020)
							Huang and Li, (2016)
							Song et al. (2018)
Glycyrrhizin	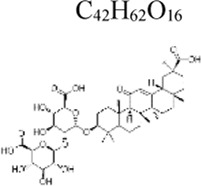 C_42_H_62_O_16_	Suppressing proinflammatory cytokines and apoptosis	Gancao (licorice root)	Rats: CLP, NR8383 cells: LPS 1 μg/mL or HMGB1 1 μg/mL	Rats:10 mg/kg, NR8383 cells: 10,50, 100 μg/mL	HMGB1↓, RAGE/TLR4 ↓ MAPK ↓ NF-κB ↓	Zhao et al. (2017)
Triptolide	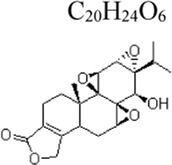 C_20_H_24_O_6_	Anti-inflammatory	Tripterygium wilfordii Hook.F	HUVECs: LPS 1 μg/mL	HUVECs: 25, 50, 100 nM	NF-κB ↓	Song C et al. (2019)
Artemisinin	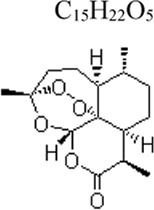 C_15_H_22_O_5_	Anti-inflammatory; Improving cognitive impairments and attenuating neuronal damage and microglial activation	artemisinin	Mice: LPS 750 μg/kg, BV2 cells: LPS 100 ng/mL	Mice: 30 mg/kg, BV2 cells: 40 μΜ	AMPKα1 ↑, NF-κB ↓	Lin S.P et al. (2021)
Ginsenoside Rg1	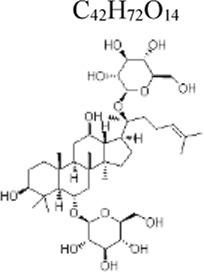 C_42_H_72_O_14_	Suppressing inflammation and apoptosis	ginseng	Mice: LPS 5 mg/kg, NRCMs cells: LPS 1 μg/mL	NRCMs cells: 20 μM	TLR4/NF-kB/NLRP3 ↓	Luo et al. (2020)
Apigenin	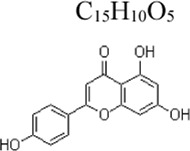 C_15_H_10_O_5_	Enhancing the antioxidant ability and decreasing the production of inflammatory cytokines	parsley, chamomile and propolis	Mice: D-GalN 700 mg/kg, LPS 20 μg/kg	Mice: 100, 200 mg/kg	Nrf-2 ↑, PPARγ ↑ NF-κB ↓	Zhou et al. (2017)
Salidroside	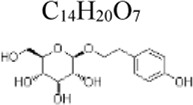 C_14_H_20_O_7_	Suppressing myocardial lipid peroxidation and inhibiting inflammatory cytokines	Rhodiola rosea	Rats: LPS 15 mg/kg, H9C2 cells: LPS 4 μg/mL (Chen L et al., 2017); Mice: CLP, RAW264.7 cells: LPS 1 μg/mL (Lan et al., 2017); HUVECs: LPS 10 μg/mL (You et al., 2021)	Rats: 20, 40 mg/kg, H9C2 cells: 20, 40 µM (Chen L et al., 2017) Mice:20, 40 mg/kg, RAW264.7 cells: 30, 60, 120 μM(Lan et al., 2017) HUVECs: 50 μM(You et al., 2021)	NF-κB ↓, PI3K/Akt/mTOR ↓; SIRT1 ↑, HMGB1 ↓, NLRP3↓	Chen L et al. (2017)
							Lan et al. (2017)
							You et al. (2021)
Baicalein	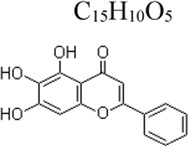 C_15_H_10_O_5_	Suppressing the ROS level and pro-inflammatory cytokines	root of Scutellaria baicalensis Georgi	BV-2 cells: LPS 0.1 μg/mL (Yan et al., 2020); THP-1 and RAW264.7 cells: LPS 1 μg/mL and ATP 5 mM (Luo et al., 2017)	BV-2 cells: .5, 1, 2, 4 µM(Yan et al., 2020) THP-1 and RAW264.7 cells: 10, 25, 50 µM(Luo et al., 2017)	ROS ↓, COX2 ↓, NF-κB ↓; TLR4/MyD88 MD-2/TLR4 complex ↓, NLRP3 ↓	Yan et al. (2020)
							Luo et al. (2017)
Resveratrol	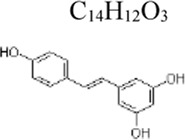 C_14_H_12_O_3_	Anti-inflammation and anti-apoptotic; Improving in vascular relaxation reactivity	mulberries, peanuts, and grape skins	Mice: CLP, MH-S cells: LPS 150 μg/mL (Yang et al., 2018); PBMC: LPS 100 ng/mL (Wang B et al., 2020); Mice: CLP (Zhang Z.S. et al., 2019)	Mice: 40 mg/kg, MH-S cells: 10 µM(Yang et al., 2018) PBMC: 40 µM(Wang B et al., 2020) Mice: 5,10 mg/kg (Zhang Z.S. et al., 2019)	VEGF-B ↑ NF-κB ↓, SphK ↓, ERK1/2 phosphorylation ↓ MyD88 ↓; Rac-1 ↓, HIF-1α ↓	Yang et al. (2018)
							Wang B et al. (2020)
							Zhang Z.S. et al. (2019)
Paeonol	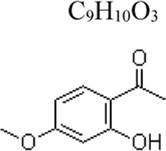 C_9_H_10_O_3_	Inhibiting the inflammatory response; Promoting the phagocytic ability of macrophages	moutan cortex	Mice: CLP, RAW264.7 cells: LPS .2 μg/mL (Mei et al., 2019); Mice: LPS .2 mg/kg, RAW264.7 cells: LPS .2 μg/mL (Miao et al., 2020)	Mice: 120 mg/kg, RAW264.7 cells: 1 mM(Mei et al., 2019); Mice: 80 mg/kg, RAW264.7 cells: 600, 1000 nM(Miao et al., 2020)	miR-339-5p ↑, HMGB1 ↓, IKK-β↓, P53 ↓	Mei et al. (2019)
							Miao et al. (2020)
6-Gingerol	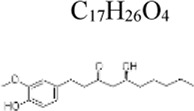 C_17_H_26_O_4_	Inhibiting inflammasome formation and pyroptosis	ginger rhizome	Mice: CLP, RAW264.7 and BMDM cells: LPS 100 ng/mL and ATP 5 mM(Zhang F.L. et al., 2020); Mice: CLP, RAW264.7 cells: LPS 1 μg/mL and ATP 5 mM(Hong et al., 2020)	Mice: 20 mg/kg, BMDMs: 8μM, RAW264.7: 4 μM(Zhang F.L. et al., 2020)	MAPK ↓, NLRP3 ↓, Nrf2 ↑	Zhang F.L. et al. (2020)
					Mice: 40 mg/kg, RAW264.7 cells: 8 μM(Hong et al., 2020)		Hong et al. (2020)
Berberine	C_20_H_18_NO_4_ 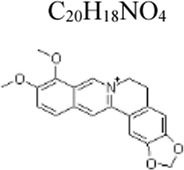	Inhibiting gluconeogenesis, insulin resistance and proinflammatory molecule release; Lowering gut-vascular barrier hyperpermeability	Coptis chinensis	Rats: CLP, HepG2 and rat intestinal microvascular endothelial cells: LPS 100 ng/mL (Li Y et al., 2020); Rats: CLP, RIMECs: LPS 50 ng/mL (He et al., 2018); Mice: LPS 5 mg/kg (Xu et al., 2021); Rats: LPS 10 mg/kg (Chen et al., 2021); RAW264.7 cells: LPS 100 ng/mL (Zhang H et al., 2017)	Rats:25, 50, 100 mg/kg, HepG2 and rat intestinal microvascular endothelial cells: 5, 10, 20 μM(Li Y et al., 2020); Rats: 25, 50 mg/kg, RIMECs: 10, 20 µM(He et al., 2018); Mice: i.p 1, 2 mg/kg and inh .1, .2 mg/mL (Xu et al., 2021); Rats: 50 mg/kg (Chen et al., 2021), RAW264.7 cells: 1, 2.5, 5 μM(Zhang H et al., 2017)	ApoM/S1P ↑ Wnt/beta-catenin ↑ TLR4/NF-κB ↓ JAK2/STAT3 ↓; SIRT1 ↑	Li Y et al. (2020)
							He et al. (2018)
							Xu et al. (2021)
							Chen et al. (2021)
							Zhang H et al. (2017)
Cordycepin	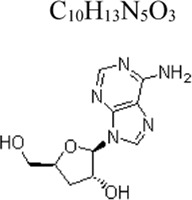 C_10_H_13_N_5_O_3_	Alleviating inflammation	Cordyceps sinensis	Mice: LPS 30 mg/kg (Qing et al., 2018); RAW264.7 cells and THP-1 cells: LPS 100 ng/mL (Yang J et al., 2017)	Mice: 1, 10, 30 mg/kg (Qing et al., 2018); RAW264.7 cells: 6.25, 12.5, 25, 50 μmol/L (Yang J et al., 2017)	Nrf2 ↑, HO-1 ↑; NLRP3 ↓, ERK1/2 ↓, COX2 ↓	Qing et al. (2018)
							Yang J et al. (2017)
Emodin	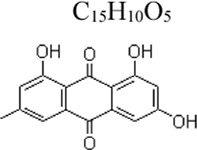 C_15_H_10_O_5_	Ameliorating hypercoagulation and fibrinolytic inhibition; Inhibiting inflammatory reaction; Anti-neuroinflammatory	Radix rhizoma Rhei	Mice: LPS 40μL, 4 mg/mL inhale (Liu B et al., 2020); Microglia cells: LPS 1 μg/mL (Park et al., 2016)	Mice: 5,10, 20 mg/kg (Liu B et al., 2020); Microglia cells: 40 μM(Park et al., 2016)	NF-κB and p65 DNA binding activity↓; AMPK/Nrf2 ↑; NF-κB ↓, AP-1↓, STAT ↓, MAPK ↓	Liu B et al. (2020)
							Park et al. (2016)

## 5 Post-sepsis immune suppression and fuzheng treatment strategy of TCM

There are many concepts recognizing the critical state of patients who are survived from early death, such as chronic critical illness (CCI), compensatory anti-inflammatory response syndrome (CARS), and persistent inflammation, immune suppression, and catabolism syndrome (PICS), among which immune suppression are a common state and has been implicated as a predisposing factor for the secondary nosocomial infections and increased mortality, though the identification of these concepts remains to be further clarified (Torres et al., 2022). Immunotherapy such as granulocyte-macrophage colony stimulating factor (GM-CSF) and granulocyte-colony stimulating factor (G-CSF) aiming to promote restoration of normal lymphocyte numbers and function, and/or restore mature functional myeloid populations achieves great improvement in preventing secondary infection, but failed to demonstrate any significant improvements in 28-day mortality and long-term outcomes (Bo et al., 2011; Darden et al., 2021).

According to the basic theory of TCM, there are two strategies for the treatment of infectious diseases and followed critical state—Quxie and Fuzheng. The function of TCM on inhibiting inflammatory response during the early sepsis refers to the Quxie strategy that means blocking of factors leading to disease. In contrast, Fuzheng strategy agree with the therapy enhancing the anti-disease capacity of the body, which focus on the enhancement of anti-infectious immunity. Innate and adaptive immune cells are both the targets of TCM herbs. Dendritic cells are the main professional antigen-presenting cells, whose function drives the activation of macrophage and antigen-specific T-cells (Lin W et al., 2021). Accumulating evidence shows that the extracts or active monomers of TCM herbs, such as cordyceps sinensis, ganoderma lucidum, astragalus mongholicus. etc. can act as the adjuvant to promote the maturation, pro-inflammatory cytokine production and function of DCs, therefore enhancing the immune responses against tumor and infection (Li et al., 2015a). It is worth recalling that astragalus, especially the astragalus polysaccharide may be the representative of TCM herbs for immunity enhancement, whose function varies from humoral to cellular immune responses (Sultan et al., 2014; Deng et al., 2022). Though there is a lack of direct evidence, studies about the immune deficiency diseases and cancer suggested the potential function of immune enhancement herbs on sepsis related immune suppression. Except for the direct inhibition of tumor, immunity-enhancing capacity plays a vital role in the anticancer activity of TCM herbs (Wang S et al., 2020; Wang Y et al., 2020) For example, shenqi fuzheng injection, mainly consist of ginsen and astragalus, could promote NK and T helper cells proliferation and function, therefore enhancing the effects of chemotherapy drugs and decreasing adverse events in cancers (Dong et al., 2010; Li et al., 2015b; Yang Y et al., 2017). Results of randomized clinical trials and real-world data reveals that integrating Fuzheng TCM herbs and anti-retroviral therapy promotes long-term reconstitution of the immune system and significantly improved the survival periods of AIDS patients (Zou et al., 2016; Tao et al., 2021; Jin et al., 2022).

## 6 Discussion and perspectives

Sepsis is a common severe complication of patients with infection, severe trauma, shock, burns, etc. With the development of public health policy, infection-prevention efforts reduce sepsis incidence. Diversified treatment and multiple organ support therapy contribute to the reduced mortality in past years. However, sepsis remains a major cause of health loss worldwide with high health-related burden, especially in Asia and Africa (Rudd et al., 2020). The problems facing the effective antibiotics-based combination therapies are the ever-growing antibiotic resistance (Reynolds et al., 2022) and the potential risk of drug-induced liver injury, enteric dysbacteriosis, and fungal infection (Kwon et al., 2022), which may further aggravate organ dysfunction (Meng et al., 2017). Therefore, more treatment strategy for sepsis is needed.

TCM established a complete system of diagnosis and treatment for pandemic and endemic diseases, possessing a well-documented history of treating infectious diseases and clinical practice, such as Treatise on Febrile and Miscellaneous Disease (“Shanghan Zabing Lun” in Chinese) and Detailed Analysis of Epidemic Warm Diseases (“Wenbing Tiaobian” in Chinese). Given the complexity of the chemical components of traditional Chinese herbs and the ambiguity of the effective components, research has been devoted to the separation of the active components of prescriptions and single traditional Chinese herb for cell or animal study. This review summarizes the function and mechanism of TCM compounds, active monomers (terpenoids, flavonoids, polyphenols, alkaloids), and acupuncture on sepsis treatment, which varies from anti-inflammation, anti-oxidation, anti-mitochondrial dysfunction, regulating apoptosis and autophagy etc. Because of the lack of clinical evidence, an important issue that is not covered in this review is whether the potential side effects of TCM on related organs would aggravate the multi-organ injury in patients with sepsis. Indeed, a study reveals that TCM herbs and dietary supplements were the leading causes of drug-induced liver injury (DILI) in mainland China (Shen et al., 2019), which attracts much attention. However, researchers pointed that implicated drug categories adopted in this study might seriously influence the reliability of conclusions (Cong et al., 2019). A latest study screened 94,593 DILI reports from 308 medical centers across the China mainland between 2012 and 2016, and found that TCM herbs only accounted for 4.5% of the DILI reports (Wang et al., 2022). The main challenge facing the application of TCM for the treatment of sepsis in drug safety aspect is how to identify the scattered categories of drug that are potentially harmful to the organ function, and the assessment of risk/benefit ratio.

In recent years, conventional molecular biological studies, network pharmacology prediction, molecular docking analysis, and visualization analysis reveal the widely potential targets of TCM compounds and active monomers in infectious diseases (Huang et al., 2021; Zhou et al., 2021; Li et al., 2022), and in this state lack of standardized and large-scale clinical studies counts in limiting clinical translation value. In addition, targeting the activation of the immune system and related imbalance of pro-inflammation and anti-inflammation is the main mechanism that studies of sepsis focus on, while the protection of organs and prevention of sequelae such as sepsis-associated encephalopathy, ICU-acquired weakness, sepsis-induced cardiomyopathy, etc., are partly neglected. Multidisciplinary research is needed to explain the scientific connotation of compound compatibility of anti-sepsis TCM recipes.

## Glossary

AChE: Acetylcholinesterase
AKI: Acute kidney injury
AKT: Protein kinase B
ALI: Acute lung injury
AP-1: Activator protein-1
ApoM: Apoliprotein M
AQP: Aquaporin
ARDS: Acute respiratory distress syndrome
ASC: Apoptosis associated speck like protein containing CARD
AST: Aminotransferase
BALF: Bronchoalveolar lavage fluid
Bcl-2: B cell lymphoma-2
BMDMs: Bone marrow-derived macrophages
CARS: Compensatory anti-inflammatory response syndrome
CAT: Catalase
CCI: Chronic critical illness
CK-MB: Creatine kinase-MB
CLP: Cecal ligation and puncture
cMLC1: cardiac myosin light chain-1
CNKI: China National Knowledge Infrastructure
COX-2: Cyclooxygenase 2
cTnI: cardiac troponin I
CXCR2: CXC motif chemokine receptor 2
DCQD: dachengqi decoction
eNOS: endothelial nitric oxide synthase
ER: endoplasmic reticulum
ERK: extracellular regulated protein kinase
GPx: glutathione peroxidase
GR: glutathione reductase
GRK2: G protein-coupled receptor kinase-2
GSDMD: gasdermin D
GSK3-β: glycogen synthase kinase-3β
GST: glutathione-S-transferase
HIF-1α: hypoxia-inducible factor 1α
HLA-DR: human leukocyte antigen DR
HLJDD: huanglian jiedu decoction
HMGB1: high mobility group box 1
ICAM-1: intercellular adhesion molecule-1
I-FABP: intestinal fatty acid-binding protein
IL-1: interleukin 1
iNOS: inducible nitric oxide synthase
IRF-3: interferon regulatory factor 3
JAK: Janus kinase
JAM-C: junctional adhesion molecule C
LDH: lactate dehydrogenase
LPS: lipopolysaccharide
MAPK: mitogen activated protein kinase
MD2: myeloid differentiation factor 2
MDA: malondialdehyde
MDSCs: myeloid-derived suppressor cells
MEK: mitogen-activated protein kinase repalmitoylated
MKK4: mitogen-activated protein kinase kinase 4
MMP-9: matrix metalloproteinase 9
MPO: myeloperoxidase
mTOR: mechanistic target of rapamycin kinase
MyD88: myeloid differentiation factor 88
NETs: neutrophil extracellular trap
NF-κB: nuclear factor-κB
NLRP3: NOD-like receptor family pyrin domain containing 3
NO: nitric oxide
Nrf-2: nuclear factor erythroid 2-related factor 2
PAMPs: pathogen-associated molecular patterns
PCT: procalcitonin
PGE2: prostaglandin E2
PI3K: phosphatidylinositol 3-kinase
Pink1: PTEN-induced kinase 1
PKM2: pyruvate kinase M2
PPARγ: peroxisome proliferator-activated receptor gamma
RAC-1: Rac family small GTPase 1
ROS: reactive oxygen species
S1P: Sphingosine-1-Phosphate
SAP: severe acute pancreatitis
SDH: succinate dehydrogenase
SFI: Shenfu injection
Sirt1: Sirtuin1
SMI: shengmai injection
SOD: superoxide dismutase
SOFA: sequential organ failure assessment
STAT: signal transducer and activator of transcription
TAB1: TAK1-binding protein1
TCM: traditional Chinese medicine
TIR: interleukin-1 receptor
TIRAP: TIR domain-containing adaptor protein
TKA1: TGF-β-activated kinase1
TLR4: Toll-like receptor 4
TNF-α: tumor necrosis factor alpha
TRAF6: TNF receptor associated factor 6
TRIF: TIR-domain-containing adapter-inducing interferon-β
TRPM7: transient receptor potential melastatin 7
TSA: Tanshinone IIA
VCAM1: vascular cell adhesion molecule 1
VEGF: vascular endothelial growth factor
VEGF-B: vascular endothelial growth factor B
XBJI: xuebijing injection
XJDHD: xijiao dihuang decoction
ZO-1: zonula occludens-1
